# Healthy Nutrition as the Key Reference in Special Diets, Quality of Life, and Sustainability

**DOI:** 10.3390/nu16172906

**Published:** 2024-08-30

**Authors:** António Raposo, Renata Puppin Zandonadi, Raquel Braz Assunção Botelho

**Affiliations:** 1 CBIOS (Research Center for Biosciences and Health Technologies), Universidade Lusófona de Humanidades e Tecnologias, Campo Grande 376, 1749-024 Lisboa, Portugal; 2 Department of Nutrition, Faculty of Health Sciences, University of Brasilia, Brasilia 70910-900, Brazil; renatapz@unb.br (R.P.Z.); raquelbotelho@unb.br (R.B.A.B.)

Healthy nutrition is considered a key factor in special diets, enhanced quality of life, and sustainability. Nutrition involves eating, which concerns how people relate to food in different contexts, with their food choices influenced by biological, social, cultural, economic, and psychological factors, as well as food availability and access to food [1,2,3]. Adopting dietary patterns such as the Mediterranean diet and plant-based diets is associated with the optimal intake of some nutrients, the prevention and management of non-communicable chronic diseases and cancer, and health promotion and sustainability [4,5,6,7,8,9].

Eating decisions can be conscious or subconscious and extend beyond basic physiological and nutritional requirements. Despite being a long-standing international human right, not everyone has access to adequate food; for instance, individuals from low-income families, those who adopt different dietary patterns, and people with dietary restrictions face challenges regarding food availability. Aside from these aspects affecting food choices, people with dietary restrictions are also subject to constraints on food consumption for disease prevention or treatment. Additionally, some individuals refrain from consuming certain types of foods for ethical, moral, religious, health, and environmental reasons. Health-related conditions (such as celiac disease, food allergies, surgeries, and others) may impose lifelong or temporary food restrictions that might be challenging and negatively affect the quality of life, diet quality, and sustainability; thus, health professionals should provide recommendations and guidance to avoid or reduce their impact [8,10,11]. In addition to health-related conditions, eating disorders and food neophobia may impact food choices and nutrient intake, thus affecting health, quality of life, and the environment [12,13]. In this sense, special diets and other dietary patterns should support an individual’s nutritional and energy needs with access to pleasing, accessible, and adequate food. A lack of proper instructions on food choices and consumption, as well as a healthy diet, may lead to the inefficacy of special diets or other dietary patterns and therefore can impact sustainability.

Since food consumption involves a complicated amalgam of ingrained habits, social norms, and acquired attitudes and feelings toward food, enhancing nutrition literacy and eating behavior through dietary interventions and strategies concerning food and nutritional education is crucial in all ages to improve nutritional and health status and avoid food waste [14,15]. Food waste is one of the leading causes of environmental decline and has an economic and social impact considering sustainability [16]. Attention to eating consumption and meal selection, variation in food and nutrient intake, avoiding monotony, and the valorization of local food production contribute to sustainability as well as health [16,17]. 
